# Quercetin Interrupts the Positive Feedback Loop Between STAT3 and IL-6, Promotes Autophagy, and Reduces ROS, Preventing EBV-Driven B Cell Immortalization

**DOI:** 10.3390/biom9090482

**Published:** 2019-09-12

**Authors:** Marisa Granato, Maria Saveria Gilardini Montani, Claudia Zompetta, Roberta Santarelli, Roberta Gonnella, Maria Anele Romeo, Gabriella D’Orazi, Alberto Faggioni, Mara Cirone

**Affiliations:** 1 Department of Experimental Medicine, “Sapienza” University of Rome, Laboratory affiliated to Istituto Pasteur Italia-Fondazione Cenci Bolognetti, 00161 Rome, Italy; 2 Translational Research Area, Regina Elena National Cancer Institute, 00128 Rome, Italy; 3 Department of Medical, Oral and Biotechnological Sciences, University “G. d’Annunzio”, 66013 Chieti, Italy

**Keywords:** quercetin, Epstein–Barr virus (EBV), STAT3, IL-6, LCLs, autophagy, SQSTM1/p62, ROS

## Abstract

The oncogenic gammaherpesvirus Epstein–Barr virus (EBV) immortalizes in vitro B lymphocytes into lymphoblastoid cell lines (LCLs), a model that gives the opportunity to explore the molecular mechanisms driving viral tumorigenesis. In this study, we addressed the potential of quercetin, a widely distributed flavonoid displaying antioxidant, anti-inflammatory, and anti-cancer properties, in preventing EBV-driven B cell immortalization. The results obtained indicated that quercetin inhibited thectivation of signal transducer and activator of transcription 3 (STAT3) induced by EBV infection and reduced molecules such as interleukin-6 (IL-6) and reactive oxidative species (ROS) known to be essential for the immortalization process. Moreover, we found that quercetin promoted autophagy and counteracted the accumulation of sequestosome1/p62 (SQSTM1/p62), ultimately leading to the prevention of B cell immortalization. These findings suggest that quercetin may have the potential to be used to counteract EBV-driven lymphomagenesis, especially if its stability is improved.

## 1. Introduction

The in vitro transformation of B lymphocytes into lymphoblastoid cell lines (LCLs) driven by Epstein–Barr virus (EBV) represents one of the most intriguing models to study tumorigenesis. Besides the role of viral proteins, the production of oxidant species, the activation of oncogenic pathways, such as nuclear factor-κB (NF-κB), mammalian target of rapamycin (mTOR), and signal transducer and activator of transcription 3 (STAT3), and their crosstalk have been shown to be involved in the immortalization process driven by EBV [1,2,3,4]. Regarding STAT3, it has been reported that its 705 Tyr phosphorylation that occurs soon after EBV infection of B cells plays a critical role in promoting the expression of oncogenic viral proteins such as latent membrane protein 1 (LMP1). LMP1 contains a STAT3 responsive element that, once expressed, sustains STAT3 activation [5]. Although the underlying molecular mechanism/s have not been completely elucidated, STAT3 is essential for LCL in vitro outgrowth, as demonstrated by the impossibility of B cells derived from patients that display hypomorphic mutation in STAT3 to give rise to LCLs [6]. Moreover, STAT3 engages a crosstalk with interleukin-6 (IL-6) in a positive feedback loop that supports B cell growth and survival [7,8] and is able to crosstalk with mTOR or NF-κB [9,10], pathways also strongly involved in B cell transformation into LCLs. Interestingly, it has been demonstrated that STAT3 is a potent negative regulator of autophagy when localized in the cytoplasm, as it interacts and inhibits protein kinase R (PKR), a kinase that plays a key role in promoting autophagy [11]. Moreover, STAT3 upregulates the expression of proteins such as Bcl-2 or Mcl-1 [12,13] that can bind and inhibit the autophagic protein Beclin-1 [12,13]. Autophagy is a multistep, degradative cellular process, whose dysregulation is strongly involved in tumorigenesis [14,15]. One of the reasons is because autophagy and, particularly, a selective form of it, i.e., mitophagy, may help cells to ride out of damaged mitochondria, the main source of oxidant species that cause DNA damage and promote cancerogenesis. Among the EBV proteins, LMP1 [16] and Epstein–Barr nuclear antigen 3B (EBNA3B) [17], essential for LCL formation, have been reported to regulate autophagy. In addition, we have previously shown that EBV blocks the autophagic flux during its replication [18,19], which suggests that the viral lytic proteins, or the pathways activated by them, may negatively regulate the last autophagic phases. As EBV replicates during the first phases of B cell infection before latency is established, we hypothesized that autophagy could be inhibited in the early phases of infection and contribute to LCL outgrowth. Indeed, this could lead to the increase of reactive oxidative species (ROS) that, besides DNA damage, may promote the activation of oncogenic pathways [20]. We have recently shown that quercetin, a flavonoid with anti-inflammatory and antioxidant properties and anti-cancer potential [21], was able to inhibit multiple pro-survival pathways activated in primary effusion lymphoma (PEL) cells, including STAT3, mTOR, and NF-κB, reducing cell survival [9]. Moreover, quercetin, likely through the inhibition of these pathways, promoted autophagy in these cells as well as in Burkitt’s Lymphoma (BL) cells [9,22], two aggressive B lymphomas strongly associated with the oncogenic gammaherpesviruses Kaposi’s Sarcoma Associated Herpesvirus (KSHV) and EBV, respectively. Based on these findings and on the previous observed anti-cancer potential of quercetin, the present study was undertaken to investigate whether this flavonoid could counteract EBV-driven B cell immortalization and to elucidate the possible molecular mechanisms involved. 

## 2. Materials and Methods

### 2.1. Cell Culture and Treatments

Peripheral blood mononuclear cells (PBMCs) obtained from healthy donors were isolated by Fycoll–Paque gradient centrifugation (Cedarlane, Burlinghton, Canada; CL5020). All subjects gave their informed consent for inclusion in this study that was conducted in accordance with the Declaration of Helsinki. The study was approved by Comitato etico Policlinico Umberto I, Roma (RIF 5787)

Briefly, whole blood was diluted 1:5 in PBS and added to an equal volume of density-gradient medium. Then, it was centrifuged at 1900 rpm for 30 min at room temperature, and PBMCs were isolated. The cells collected were washed three times in PBS supplemented with 10% FBS. For EBV immortalization of B cells, 50 × 10^6^ PBMCs were infected at multiplicity of infection (MOI) of 10 genome equivalent/cell for 2 hours at 37 °C and then seeded in 24-well plates at 1 × 10^6^/mL in 20 mL of complete RPMI 1640 supplemented with cyclosporine A (1 μg/mL) (Sigma Aldrich, St Louis, MO, USA; 30024) [23]. Then, infected and uninfected cells were treated with quercetin (Q) (Sigma Aldrich; 337951) (10 μM) or with the same amount of DMSO as a control and grown in RPMI 1640 (Thermo Fisher Scientific, Waltham, MA, USA; 21870) supplemented with 10% Fetal Bovine Serum (FBS) (Corning, NY, USA; 35-079), with l-glutamine and streptomycin (100 µg/mL) (Corning, NY, USA; 30-002) as well as penicillin (100 U/mL) (Corning, NY, USA; 25-005) in 5% CO2 at 37 °C. Quercetin was replenished every three days for all the time of culture.

In some experiments, in order to investigate autophagy, uninfected and infected cells were treated with Bafilomycin A1 (BAF) (20 nM) (Santa Cruz Biotechnology Inc., Dallas, TX, USA; sc-201550), an inhibitor of vacuolar H+-ATPase, for the last four hours.

### 2.2. Virus Preparation

B95.8, an EBV-infected lymphoblastoid cell line derived from marmoset monkey, was cultured in RPMI medium and used as a viral source [24]. To produce viral particles, 5 × 10^6^ B95.8 cells were plated in a 175 cm^2^ flask and cultured in RPMI medium to at least 80% of confluence and then were treated with phorbol 12-myristate 13-acetate (TPA) (T) (Sigma Aldrich, St Louis, MO, USA; P8139) (40 ng/mL) and sodium butyrate (B) (Sigma Aldrich; B5887) (6 mM) for 2 days. Subsequently, cell supernatants were collected after centrifugation at 29,000 rpm for 1 h and 30 minutes at 4 °C. Finally, the supernatants were discarded, and the pellets were resuspended in FBS. Aliquots were stored at −80 °C [25]. 

### 2.3. Cell Viability

A trypan blue (Sigma Aldrich; 72571) exclusion assay was performed to assess cell viability. Only cells with intact membranes can effectively exclude the dye, so dead cells with compromised membranes become stained in blue. Unstained cells (live cells) were counted by light microscopy using a Neubauer hemocytometer. 

### 2.4. Antibodies

In western blotting, we used the following primary antibodies: mouse monoclonal anti-STAT3 (1:1000) (BD Transduction Laboratories, San Jose, CA, USA; 612356), mouse monoclonal anti-phosphorylate-STAT3 (Tyr705) (1:100) (Santa Cruz Biotechnology Inc., Heidelberg, Germany; sc-8059), mouse monoclonal anti-sequestome1 (SQSTM1) (1:500) (BD Transduction Laboratories; cat. no. 610833), and rabbit polyclonal anti-LC3 (1:1000) (Novus Biologicals, Cambridge, UK; NB100-2220SS). To assess EBV infection, rabbit polyclonal anti-BRLF1 antibody (1:100) (Bioss Antibodies, Woburn, MA, USA; bs-4542R) and anti-EBNA1 antibody (1:100) (Santa Cruz Biotechnology Inc., Heidelberg, Germany; sc-81581) were used. Mouse monoclonal anti-β-actin (1:10,000) (Sigma Aldrich; A5441) (1:10,000) was used to detect β-actin, serving as a loading control. The goat polyclonal anti-mouse IgG-horseradish peroxidase (HRP, Santa Cruz Biotechnology Inc., Heidelberg, Germany; sc-2005) and anti-rabbit IgG-HRP (Santa Cruz Biotechnology Inc., Heidelberg, Germany; sc-2004) were used as secondary antibodies. All the primary and secondary antibodies were diluted in PBS-0.1% Tween 20 solution containing 3% of BSA (SERVA, Reno, NV, USA; 11943.03).

### 2.5. Western Blot Analysis

A total of 5 × 106 PBMCs were washed twice with 1X PBS and centrifuged at 1500 rpm for 5 min. Cells were lysed in a 1X RIPA buffer containing 150 mM NaCl, 1% NP-40 (Sigma Aldrich; NP40S), 50 mM Tris-HCl, pH 8, 0.5% deoxycholic acid (Sigma Aldrich, D6750), 0.1% SDS (Sigma Aldrich, 71736), protease (Sigma Aldrich; S8830) and phosphatase inhibitors (Sodium Orthovanadate; Sigma Aldrich; S6508) (Sodium Fluoride; Sigma Aldrich; S7920). Then, 15 µg of protein lysates was subjected to electrophoresis on 4–20% SDS-PAGE gradient gels (NuSep, Germantown, MD, USA; NN12-420), according to the manufacturer’s instructions. Then, the gels were transferred to nitrocellulose membranes (Bio-Rad, 162-0115) for 2 h in Tris-glycine buffer. The membranes were blocked in PBS-0.1% Tween 20 solution containing 3% BSA, probed with specific antibodies, and developed using ECL Blotting Substrate (Advansta, San Josè, CA, USA; K-12045-D20).

### 2.6. Endogenous Reactive Oxygen Species Detection

To detect ROS, uninfected or infected PBMC were treated with quercetin (Q) (Sigma Aldrich; 337951) (10 μM) up to 3 days. Endogenous ROS production was measured at different times (2, 16, 40, and 72 h) post-infection (PI). Briefly, cells were stained with 2′,7′-dichlorofluorescein diacetate (DC-FDA) (Thermo Fisher Scientific; D399), a fluorogenic dye which diffuses into the cell. Usually, DC-FDA is oxidized by ROS into 2′,7′-dichlorofluorescein, a fluorescent compound which can be detected by fluorescence spectroscopy. The cells were washed twice with 1X PBS and then were incubated at 37 °C with 10 μM DC-FDA for 15 min, as previously reported [26]. Then, the cells were washed and analyzed in FL-1 by a FACScalibur flow cytometer (BD Diagniostics, Franklin Lakes, NJ, USA). For each analysis, 10,000 events were recorded.

### 2.7. ELISA Assay

Uninfected and EBV-infected PBMCs were treated with quercetin (Sigma Aldrich; 337951) (10 μM) for the indicated times. To measure IL-6 release, ELISA (RayBiotech, Inc, Peachtree Corners, GA, USA; ELH-IL6-1) assays were performed, according to the manufacturer’s instructions. Briefly, supernatants were added in duplicate to pre-coated plates for the indicated times, and then the plates were washed three times. Finally, the samples were incubated with horseradish peroxidase-conjugated antibody, from the same kit, and the substrate tetramethylbenzidine (TMB) was added. IL-6 concentration was measured using a microplate reader (Multiskan Ex, Thermo Labsystem, L. Cagnola, 35, Abbiategrasso, Italy.), evaluating the absorbance at 450 nm. The observed values were expressed as pg/ mL.

### 2.8. Densitometric Analysis

The quantification of protein bands was performed by densitometric analysis using the Image J software [27], which was downloaded from the NIH website (http://imagej.nih.gov).

### 2.9. Statistical Analysis

Results are represented by the mean ± standard deviation (SD) of at least three independent experiments, and a two-tailed Student’s *t*-test was used to demonstrate statistical significance. Difference was considered as statistically significant when the *p*-value was at least <0.05.

## 3. Results

### 3.1. Quercetin Counteracts B Cell Immortalization into LCLs Driven by EBV

**Figure 1:**
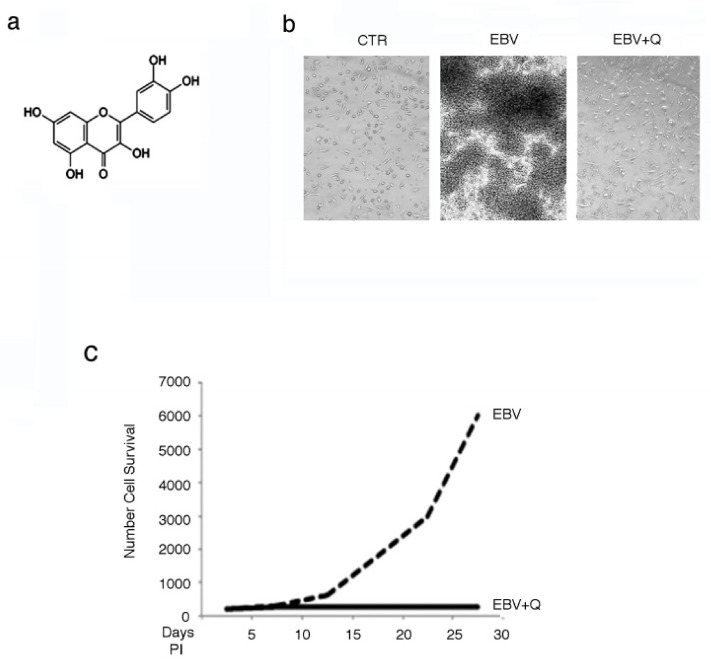
Quercetin (Q) prevents Epstein–Barr virus (EBV)-mediated B cell immortalization. (**a**) Quercetin structure, (**b**) optical microscope photos showing the inhibitory effect of quercetin on lymphoblastoid cell line (LCL) formation, (**c**) Time-dependent growth curve, as evaluated by a trypan blue exclusion assay of EBV-infected B cells grown in the presence or in the absence of quercetin. A representative experiment out of three is shown. CTR (control); PI (post-infection).

To evaluate whether quercetin could inhibit B cell immortalization driven by EBV, PBMCs were infected by EBV derived from B95-8 cells and cultured in the presence or absence of quercetin at non-toxic concentrations, as assessed in preliminary experiments (data not shown). Quercetin, whose structure is reported in Figure 1a, was replenished every three days. After four to five weeks, upon optical microscopy observations (20X), foci, typical of transformed LCLs, were visible in the EBV-infected culture, while several isolated cells were seen in quercetin-supplemented cultures (Figure 1b). As trypan blue-stained cells were not visible in the cultures of EBV-infected cells whether treated with quercetin or not, living cells were counted by light microscopy using a Neubauer hemocytometer, and a representative growth curve is reported in Figure 1c.

### 3.2. Quercetin Interrupts the Positive Feedback Loop Between STAT3 and IL-6 in EBV-Infected B Lymphocytes

**Figure 2:**
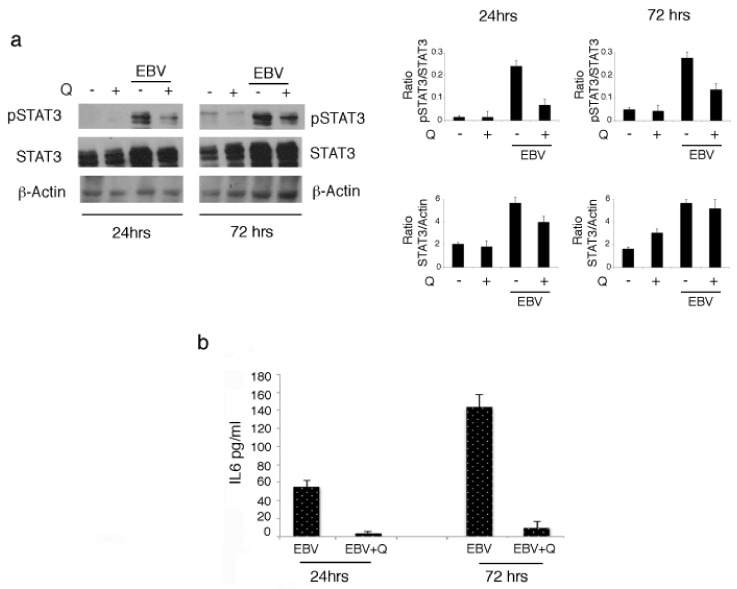
Quercetin inhibits the crosstalk between signal transducer and activator of transcription 3 (STAT3) and interleukin-6 (IL-6) in EBV-infected cells. (**a**) Western blot analysis of STAT3 705 Tyr phosphorylation evaluated following EBV infection of B cells in the presence or absence of quercetin at 24 and 72 h post-infection. Densitometric analysis was performed using Image J software, and the ratio of p-STAT3 vs. STAT3 or of STAT3 versus β-actin, was calculated. Histograms represent the mean ± SD of three independent experiments. (**b**) IL-6 release by EBV-infected cells in the presence or in the absence of quercetin at 24 and 72 h. Histograms represent the mean ± standard deviation (SD) of IL-6 release (pg/mL) from three independent experiments.

As a positive feedback loop between STAT3 and IL-6 has been reported to promote B cell growth, and several lines of evidence have indicated STAT3 as a key molecule in EBV-driven transformation of B cells into LCLs [28], we monitored STAT3 activation and IL-6 production by B cells infected by EBV at two different time points. As shown in Figure 2a,b, we found a time-dependent increase of both STAT3 705 Tyr phosphorylation and IL-6 production (Figure 2b). As previous findings have indicated that quercetin was able to inhibit STAT3 activation in gammaherpesvirus-associated B lymphoma [9], here we investigated whether it could also mediate this effect in EBV-infected B cells. The results obtained indicated that quercetin counteracted the increase of STAT3 phosphorylation (Figure 2a) as well as the increase of IL-6 production, suggesting that it was able to interrupt the positive feedback loop occurring between these molecules and thus to interfere with a mechanism required for EBV-mediated B cell immortalization.

### 3.3. Quercetin Restores the Autophagic Flux Blocked by EBV Infection 

**Figure 3:**
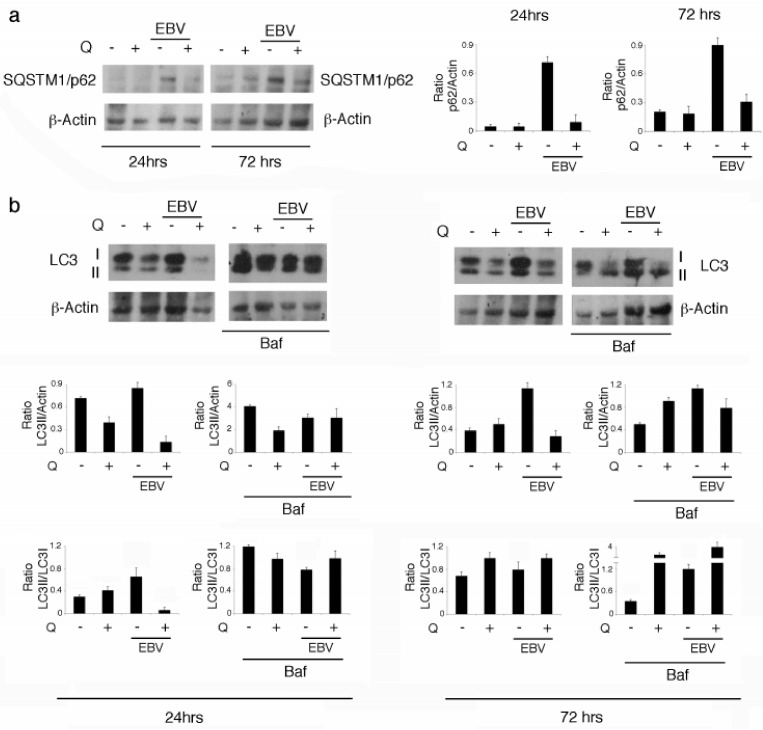
The autophagic flux was blocked by EBV and restored by quercetin. (**a**) Sequestosome1/p62 (SQSTM1/p62) expression level was investigated following EBV infection of B cells treated or not with quercetin at 24 and 72 h post-infection. (**b**) Microtubule-associated protein 1 light chain 3-I/II (LC3-I/II) as evaluated by western blot analysis in EBV-infected B cells, treated or not with quercetin, in the presence or in the absence of Bafilomycin 1 (Baf), at 24 and 72 h post-infection. Densitometric analysis was performed using Image J software and the ratio of SQSTM1/p62 or LC3-II vs. b-actin was calculated. Histograms represent the mean ± SD of three independent experiments.

Quercetin can induce autophagy in gammaherpesvirus-infected lymphoma cells [9,22], a cellular process inhibited by EBV during its replicative cycle [18]. Given that EBV replicates soon after infection of B cells, before the establishment of viral latency, we hypothesized that autophagy could be inhibited at this time. Autophagy inhibition could also contribute to EBV-mediated STAT3 activation and IL-6 production (Figure 2a,b), according to previous findings [14]. After 24 and 72 h of infection, we observed that SQSTM1/p62 progressively accumulated in EBV-infected cells (Figure 3a), suggesting a reduction of autophagy. Interestingly, while quercetin reduced STAT3 and IL-6 production (Figure 2a,b), it also counteracted SQSTM1/p62 accumulation (Figure 3a), likely by restoring autophagy inhibited by EBV. To demonstrate that quercetin was able to promote a complete autophagic flux in EBV-infected B cells, we analyzed microtubule-associated protein 1 light chain 3-I/II (LC3-I/II) expression levels in the presence or in the absence of bafilomycin (Baf), as LC3-II is formed and degraded during a complete autophagic process. The results shown in Figure 3b indicate that LC3-II expression levels, which did not change in the presence or in the absence of Baf in EBV-infected B cells, increased in the presence of Baf following quercetin treatment. These results indicate that autophagy was blocked by EBV infection and restored by quercetin in B cells.

### 3.4. Quercetin Counteracts ROS Increase But Does Not Affect EBV’s BRLF1 and EBNA1 Expression in EBV-Infected B Cells

**Figure 4:**
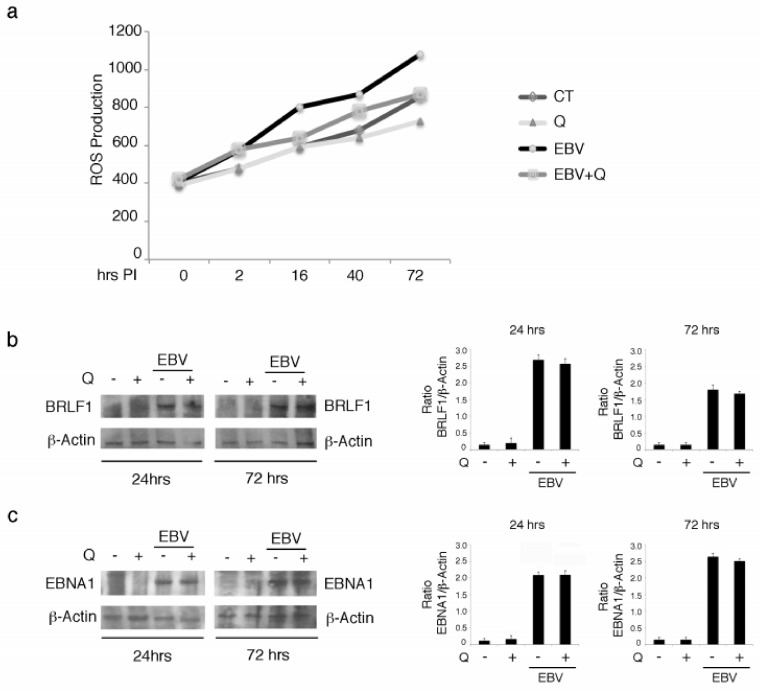
Quercetin reduces reactive oxidative species (ROS) but does not affect BRLF1 expression in EBV-infected B lymphocytes. (**a**) Time-dependent increase of intracellular ROS evaluated by 2′,7′-dichlorofluorescein diacetate (DC-FDA) staining and FACS analysis. The mean of fluorescence intensity is reported on the Y-axis (**b**). (**c**) BRLF1 and EBNA1 expression levels as evaluated by western blot analysis following EBV infection of B cells, with or without treatment with quercetin. Densitometric analysis was performed using Image J software, and the ratio of BRLF1 vs. b-actin was calculated. Histograms represent the mean ± SD of three independent experiments.

As autophagy may represent a route for ROS degradation, and quercetin is known to have antioxidant properties, we assessed ROS levels in EBV-infected B cells in the presence or in the absence of quercetin. We found a time-dependent increase of ROS following viral infection, which was reduced by quercetin (Figure 4a). As ROS have been shown to strongly contribute to EBV-driven B cell transformation and may also sustain STAT3 activation [27,28], their reduction could represent an important mechanism involved in quercetin prevention of LCL formation. Finally, we investigated whether quercetin could affect EBV protein expression. The expression levels of the lytic and latent proteins BRLF1 and EBNA1 were evaluated in virus-infected B cells in the presence or in the absence of quercetin. As shown in Figure 4b,c, quercetin did not modify either BRLF1 or EBNA1 expression after 24 or 72 h of infection, suggesting that it halted EBV-mediated immortalization of B lymphocytes by affecting cellular pathways/processes rather than by reducing the expression of viral proteins.

## 4. Discussion

One of the main goals in anti-cancer therapy is to discover new cytotoxic treatments that are effective against cancer cells while safe for normal cells, especially those belonging to the immune system. These characteristics are held by flavonoids or capsaicinoids, natural products that display antioxidant, anti-inflammatory, and anti-cancer properties [29,30]. One of the most studied flavonoids is quercetin, a molecule widely diffused in the plant kingdom, that was shown to be cytotoxic against a variety of cancer types [31]. It is indeed able to inhibit multiple oncogenic pathways such as STAT3, is constitutively activated in tumor cells, and can potentiate the immune response [9,22]. However, the cytotoxic effect of quercetin against EBV-infected gastric cancer cells has been reported to occur also independently of STAT3 inhibition [32]. It is interesting to investigate the role of flavonoids in cancer prevention, as their antioxidant properties could be promising in reducing cancer risk. This study used a model of viral tumorigenesis induced by EBV to assess quercetin’s tumor prevention capacity. We showed for the first time that quercetin prevented EBV-driven LCL immortalization, suggesting that besides being effective in reducing the survival of gammaherpesvirus-associated B lymphoma cells, it may also hold the potential to prevent EBV-lymphoproliferative malignancies that mainly arise in immune-deficient patients [33]. However, studies which modify the structure of quercetin or associate it with other molecules in order to increase its stability are necessary to improve our understanding of its function. With respect to the underlying mechanisms, in this study, we found that quercetin counteracted the activation of molecules and pathways that recapitulate the induction of EBV-mediated tumorigenesis, as it counteracted STAT3 705 Tyr phosphorylation, the production of IL-6, and the increase of ROS, key molecules in B cell immortalization. Another new finding of this study is that EBV, in correlation with STAT3 activation [11], reduced autophagy during the first phases of infection of B cells, and this effect was also counteracted by quercetin. Although autophagy inhibition has been reported to favor tumorigenesis, i.e., by reducing damaged mitochondria and ROS elimination, whether it could be reduced in B cells following EBV infection has not been previously shown. Interestingly, a previous study has reported that the highly proliferating fraction of LCLs needs a balanced level of autophagy to produce the biosynthetic intermediates necessary for cell growth [34]. Here, we found that by restoring autophagy, quercetin prevented SQSTM1/p62 accumulation, as this protein is mainly degraded through this route. Even if SQSTM1/p62 may also activate apoptosis, its role in promoting tumorigenesis is well documented, and, for example, it may activate NF-κB [35]. For this reason, it has been reported that “autophagy prevents tumorigenesis by eliminating SQSTM1/p62” [36]. Of note, besides the reduction of autophagy, the upregulation of p62 occurring in B cells soon after EBV infection could correlate with the increase of ROS that may stabilize NRF2, a transcription factor promoting SQSTM1/p62 transcription [9,22,37]. Therefore, by reducing ROS, quercetin could further lead to the reduction of p62 expression level. Interestingly, a recent study has directly linked p62 to EBV-induced cancerogenesis as it has shown that p62 accumulation destabilizes DNA damage repair (DDR) proteins and promotes genome instability in EBV-transformed B cells [38]. Thus, we hypothesize that the increase of p62 could play a key role in EBV-driven B cell immortalization, although additional studies aimed at completely deleting p62 will help to better clarify its role in this process. Indeed, the partial reduction of p62 achieved by siRNA experiments seems to give opposite results and further reduce DDR protein stabilization [38]. 

## 5. Conclusion

In conclusion, this study demonstrates for the first time that quercetin was able to counteract EBV-driven immortalization of B cells and LCL outgrowth. This effect seems to occur by several means, i.e., quercetin interrupted the crosstalk between IL-6 and STAT3, promoted autophagy, and reduced ROS levels and p62 accumulation. These findings suggest the need for greater efforts aimed at discovering new strategies that may increase the stability of this molecule and exploring its potential in the prevention of EBV-driven lymphomagenesis.
